# Glucose-dependent insulinotropic polypeptide stimulates post-absorptive lipid secretion in the intestine

**DOI:** 10.3389/fphys.2025.1549392

**Published:** 2025-04-04

**Authors:** Rong Wang, Muhammad Saad Abdullah Khan, Kundanika Mukherjee, Murooj Ghanem, Changting Xiao

**Affiliations:** Department of Anatomy, Physiology and Pharmacology, College of Medicine, University of Saskatchewan, Saskatoon, SK, Canada

**Keywords:** intestine, glucose, glucose-dependent insulinotropic peptide, chylomicron, triglycerides

## Abstract

It is increasingly recognized that the intestine can retain a portion of dietary fats for secretion during the post-absorptive state, which has strong implications in metabolic diseases. The regulatory mechanisms of gut lipid storage and release are not well defined. Previous studies showed that the intestine releases locally stored fats in response to several stimulatory cues, such as glucose delivered into the intestinal lumen. It remains unknown how the intestine responds to nutrient signals in this phenomenon. Here we tested the effects of intravenous glucose delivery on intestinal lipid output during the post-absorptive state in mesenteric lymph duct cannulated rats. Compared with intraduodenal glucose delivery, intravenous glucose did not stimulate intestinal lipid output. Intraduodenal glucose was also associated with increases in blood levels of metabolic hormones, among which glucose-dependent insulinotropic peptide (GIP) levels were significantly higher at timepoints corresponding to increased lipid output than in intravenous glucose. Intraperitoneal GIP administration *per se* robustly stimulated intestinal lipid output. These results support a mechanism that involves glucose sensing at the apical side of the enterocytes and GIP as a potent stimulus for the release of lipid storage from the intestine.

## Introduction

Intestinal handling of dietary fats plays important roles in maintaining energy and lipid homeostasis and overall health. Dysregulated lipid handling in the intestine is closely related to the development of metabolic diseases (Wit et al., 2022). It is well known that dietary fats are mostly packaged into lipoprotein particles (chylomicrons) in intestinal absorptive cells (enterocytes) for secretion. A portion of dietary fats are also retained in the intestine for prolonged time into the post-absorptive state. Such “enteral” lipid stores are thought to include cytoplasmic lipid droplets in enterocytes and chylomicrons in locations beyond enterocytes, such as intercellular spaces, the lamina propria, and the mesenteric lymphatic vasculature, and are secreted upon receiving certain stimulatory cues (Xiao et al., 2019a; Syed-Abdul et al., 2022; Ghanem et al., 2022; Xiao et al., 2019b). Importantly, lipid storage and release in the intestine is associated with metabolic health, thus insulin resistant humans have reduced storage capacity, which contributes to their postprandial lipemia, a risk factor for atherosclerotic cardiovascular disease (Jacome-Sosa et al., 2021). The regulatory mechanisms of intestinal lipid storage and release are not well defined. Better understanding of this phenomenon may help develop therapeutic approaches towards dyslipidemia and atherosclerotic cardiovascular diseases.

Release of enteral stored lipids during the post-absorptive state have been shown to respond to several stimulatory factors in humans. One such stimulus is sequential meal, where ingestion of a meal leads to early appearance of chylomicron triglycerides (TGs) that originate from an earlier fatty meal, i.e., the “second meal effect” (Evans et al., 1998; Fielding et al., 1996). Sham fat feeding, i.e., tasting but not ingesting of fats, also elicits similar effects (Chavez-Jauregui et al., 2010). The gut hormone glucagon-like peptide-2 (GLP-2) promotes post-absorptive chylomicron secretion, primarily mobilizing chylomicrons from non-enterocyte locations (Dash et al., 2014). Finally, ingestion of glucose releases lipids stored in the intestine, primarily by directing cytoplasmic lipid droplets towards chylomicrons synthesis (Xiao et al., 2019c; Robertson et al., 2003).

Several key questions remain regarding how nutrient signals, such as glucose, dictate enterocytes in their secretion of chylomicrons. Besides glucose ingestion in humans, glucose delivery into the duodenum also releases stored lipids from the intestine in rats (Stahel et al., 2019). This and oral ingestion of glucose in humans both support that the enterocytes respond to glucose at the intestinal lumen. Enterocytes are polarized cells. They not only absorb dietary nutrients from the intestinal lumen (apical side), but also take up substances from the basolateral side. For instance, basolateral uptake of lipid substrates contributes to enterocyte intracellular metabolism (Li et al., 2019; Storch et al., 2008). Basolateral glucose uptake also modulates cholesterol metabolism in enterocytes (Grenier et al., 2013). In humans, intestinal lipoprotein synthesis responds to glucose supply into both the intestinal lumen (apical side) and the blood circulation (basolateral side) during fed state (Xiao et al., 2016; Xiao et al., 2013). A key question is whether the release of lipid stores during the post-absorptive state responds only to lumen glucose but not basolateral glucose. Glucose in the GI track stimulates the secretion of gut hormones, such as the incretin hormones glucagon-like peptide-1 (GLP-1) and glucose-dependent insulinotropic polypeptide (GIP). Metabolic hormones regulate lipid metabolism in the intestine. For example, GLP-1 inhibits intestinal lipoprotein synthesis in humans (Xiao et al., 2012) and rodents (Hsieh et al., 2010; Hoffman et al., 2022). Leptin regulates the expression of microsomal triglyceride transfer protein (MTP, a key protein in chylomicron synthesis) in the intestine (Iqbal et al., 2020), and lower intestinal lipid storage in insulin resistant humans inversely correlates with plasma leptin levels (Jacome-Sosa et al., 2021). A further question is whether glucose mobilizes lipids directly via sensing of glucose or indirectly via glucose-responsive hormones. We hypothesized that mobilization of lipid stores by glucose requires glucose sensing at the intestinal lumen and is mediated by glucose-responsive hormones.

Here, in mesenteric lymph duct cannulated rats, we provided glucose through intravenous infusion. Its effects on the release of stored lipids and the secretion of metabolic hormones were compared with glucose delivery through intraduodenal infusion. Based on differential changes in hormone profiles between treatments, we further tested GIP as a candidate intermediate mediator on lipid output in the intestine. This study for the first time demonstrates that GIP is an important hormonal regulator of post-absorptive lipid release from the intestine.

## Materials and methods

### Animals

Male Sprague-Dawley rats (8–10 weeks old) were obtained from Charles River Laboratories (Senneville, QC, Canada). Animals were housed with a 12-h light/dark cycle with *ad libitum* access to standard rodent chow (LabDiet, St. Louis, MO; Prolab® RMH 3000; calories provided by protein 26.1%, fat 14.4%, carbohydrates 59.5%) and water. The study was approved by the Animal Research Ethics Board at the University of Saskatchewan.

### Surgical procedures

Rats were surgically implanted with catheters into the mesenteric lymph duct, the duodenum, the right jugular vein, and the peritoneum as described previously with minor modifications (Stahel et al., 2019; Kohan et al., 2015; Feng et al., 2015). Briefly, under general anaesthesia, a midline incision was made in the abdomen. Following identification and dissection of the mesenteric lymph duct, a polyvinyl chloride tubing (0.50 mm ID, 0.80 mm OD) was inserted and secured with a drop of cyanoacrylate glue. A fundal incision was made in the stomach and a silicone tubing (1.02 mm ID, 2.16 mm OD) was inserted, advanced through the pylorus ∼2 cm into the duodenum, and secured by purse-string suture. A PE-50 tubing with a silastic tip was inserted into the right jugular vein, secured by sutures, before being tunnelled under the skin and exteriorized at the back of the neck. A PE-50 tubing was also placed into the peritoneum and exteriorized at the abdominal flank. After surgery, rats were housed in a modified Bollman restraint cage placed inside a custom-made recovery incubator with ventilation and temperature control to maintain ambient temperature at 26°C. Normal saline with 5% glucose was infused via the intraduodenal catheter at a rate of 3 mL/h. To simulate an overnight fast, infusion was switched to normal saline without glucose at the end of day and continued throughout the remainder of the study.

### Experimental design

#### Study 1. Examining the effects of glucose delivery routes on post-absorptive lipid output in the intestine

Following recovery and an overnight fast, rats received an intraduodenal lipid load (Intralipid 20%, 1.5 mL, Sigma-Aldrich). 5 h later, rats (n = 7–8 per group) were randomly assigned to two treatment groups to receive either intraduodenal (ID) or intravenous (IV) infusion of glucose (0.2 g in 1 mL saline, infused over 2 min). To minimize any impact of fluid administration route and volume, rats in ID group also received intravenous saline (1 mL) while rats in IV group also received intraduodenal saline (1 mL). Lymph fluid was collected into tubes on ice throughout the experiment. Blood samples were collected 30min before, and at regular intervals after treatments, into collection tubes for serum separation. The following inhibitors were added to collection tubes (per mL blood): orlistat (TCI America, 0.55 μg), aprotinin (Sigma-Aldrich, 1 μg), dipeptidyl peptidase IV inhibitor (Millipore, 10 μL), serine protease inhibitor (Roche, 1 mg), and protease inhibitor cocktail (Sigma-Aldrich, 0.1 μL).

#### Study 2. examining the effects of GIP on post-absorptive lipid output in the intestine

Following recovery and an overnight fast, rats received an intraduodenal lipid load (Intralipid 20%, 1.5 mL, Sigma-Aldrich). 5 h later, rats (n = 7–8 per group) were randomized to receive intraperitoneal infusion of either a long-acting GIP analogue (D-Ala2-GIP, 0.12 mg/kg, in 1 mL PBS; Pepceuticals Ltd., Leicestershire, UK) or placebo (PBS, 1 mL). This dose of D-Ala-GIP when administered daily modulated gene expression in adipose tissues in mice (Szalowska et al., 2011). A single injection of a third of this dose improved glucose tolerance in rats (Hinke et al., 2002). Lymph fluid was collected into tubes on ice throughout the experiment.

### Lymph TG and apolipoprotein B48 (ApoB48) assays and calculations of lymph TG and ApoB48 output

Lymph TG concentrations were determined using commercial kit (FUJIFILM, Lexington, MA). In the study where ID and IV glucose was tested, lymph ApoB48 was separated with SDS-PAGE, blotted with ApoB (A-6) primary antibody (sc-393636, Santa Cruz Biotech, US), and m-IgG Fc BP-HRP secondary antibody (sc-525409, Santa Cruz). Gels were imaged with a Bio-Rad ChemiDoc, quantified with densitometry and normalized with an internal reference sample. In the study where GIP was tested, lymph apoB48 was measured using a rat specific ApoB48 ELISA Kit (CUSABIO, US). Lymph flow rates were calculated as volume of lymph collected over time. Lymph TG output was calculated as lymph flow rate X lymph TG concentration (Stahel et al., 2019). Lymph apoB48 output was calculated as lymph flow rate X lymph apoB48 concentration.

### Analysis of metabolic hormones

Serum levels of metabolic related hormones were analysed using a Rat Metabolic Discovery Assay® Array (MRDMET12; Eve Technology, Calgary, AB). Optimal dilution for samples was established prior to final assays. The array simultaneously analyzed the following hormones: insulin, glucagon, C-Peptide 2, amylin (active), GIP (total), GLP-1 (active), ghrelin (active), leptin, pancreatic polypeptide (PP), and peptide YY (PYY).

### Statistical analysis

All the data were presented as mean ± SEM. Statistical analyses were performed using GraphPad Prism (Version 10). Time-course curves were analyzed using Two-way Repeated Measures ANOVA followed by post-hoc Tukey’s multiple comparisons test. Single endpoint measures (AUC, Total Lymph Volume 0–1 h and Total TG Output 0–1 h) were analyzed using unpaired Student’s t-test. Statistical significance was set at *p* < 0.05.

## Results

### Glucose delivery into the small intestinal lumen, but not blood circulation, mobilized lipid stores from the intestine.

**FIGURE 1:**
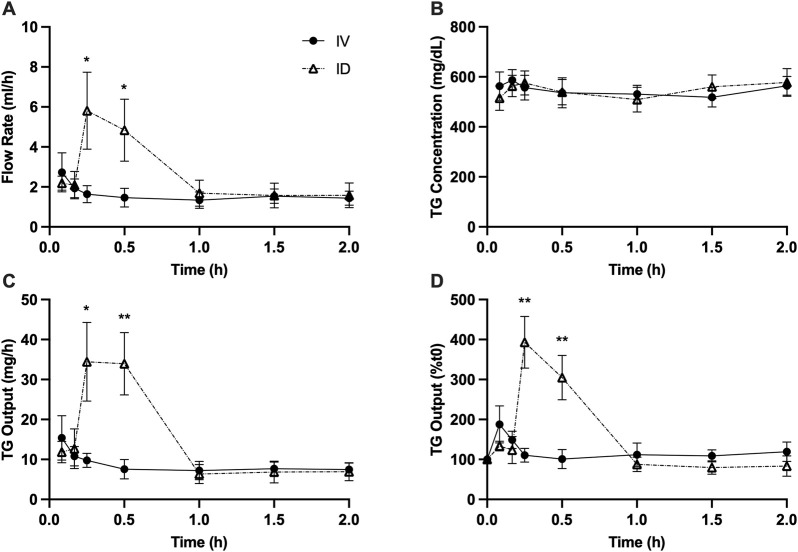
Intraduodenal (ID) but not intravenous (IV) delivery of glucose increases lymph fluid and TG output from the gut during post-absorptive state. **(A)** Lymph flow rate, **(B)** TG concentration, **(C)** TG output and **(D)** TG output expressed as % change from t = 0 during the experiment. Data are mean ± SEM. n = 7 each group. *p < 0.05, **p < 0.01 IV vs. ID.

**FIGURE 2:**
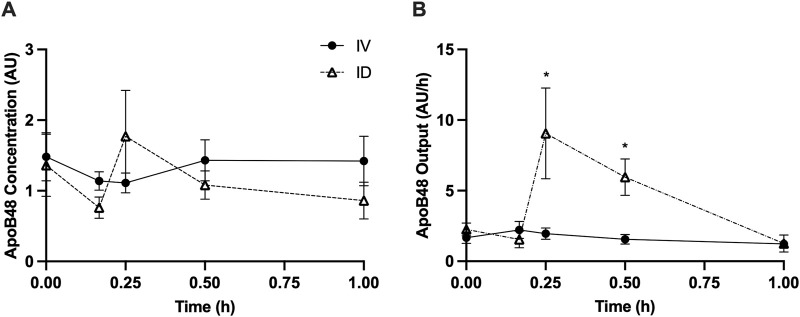
Intraduodenal (ID) but not intravenous (IV) delivery of glucose increases apolipoprotein B48 output from the gut during post-absorptive state. **(A)** Lymph ApoB48 concentration and **(B)** lymph apoB48 output during the experiment. Data are mean ± SEM. n = 7 each group. *p < 0.05 IV vs. ID.

Intraduodenal glucose infusion increased lymph flow rate from approximately 2 mL/h to 5–6 mL/h at 15 and 30 min. In contrast, this effect was not observed with intravenous glucose infusion (Figure 1A). Lymph TG concentrations were similar between treatments (Figure 1B). As a result, calculated TG output at 15 min (*p* < 0.05) and 30 min (*p* < 0.01) was significantly higher in rats received intraduodenal vs. intravenous glucose (Figure 1C). To better illustrate the magnitude of the effects, TG output was also expressed as % change from baseline, which shows that TG output increased from t = 0 by 3∼4 fold at 15 and 30 min following intraduodenal glucose but remained relatively unchanged following intravenous glucose (Figure 1D). Lymph ApoB48 concentrations were similar between treatments (Figure 2A). Lymph ApoB48 output increased from baseline at 15 and 30 min following intraduodenal glucose, but was not affected by intravenous glucose, which resulted in ApoB48 output at 15 and 30 min significantly higher following intraduodenal vs. intravenous glucose (*p* < 0.05 at both timepoints) (Figure 2B). Non-glucose controls were not included in this experiment. In the same mesenteric lymph duct cannulated rat model, ID infusion of the same amount and volume of saline did not cause significant changes in gut secretion of fluid and lipids (Stahel et al., 2019). Importantly, in Study 2 (Figures 5, 6), gut secretion of lymph fluid and lipids remained flat following PBS alone. These results therefore indicate that glucose delivery to intestinal lumen (apical side) stimulated chylomicron secretion, while glucose delivery to blood circulation (basolateral side) did not have such effects.

### Different glucose delivery routes led to differential changes in circulating levels of metabolic hormones.

**FIGURE 3:**
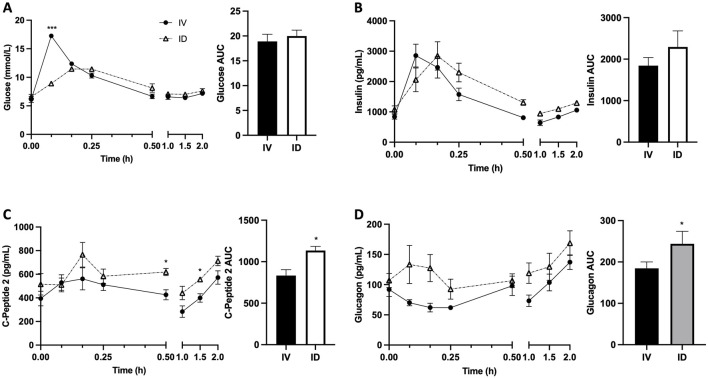
Intraduodenal (ID) and intravenous (IV) delivery of glucose lead to differences in circulating levels of glucose and glucose-responsive hormones. Serum concentrations (left) and AUC (right) of **(A)** glucose, **(B)** insulin, **(C)** C-peptide 2 and **(D)** glucagon. Data are mean ± SEM. n = 7 each group. *p < 0.05, ***p < 0.001 IV vs. ID.

**FIGURE 4:**
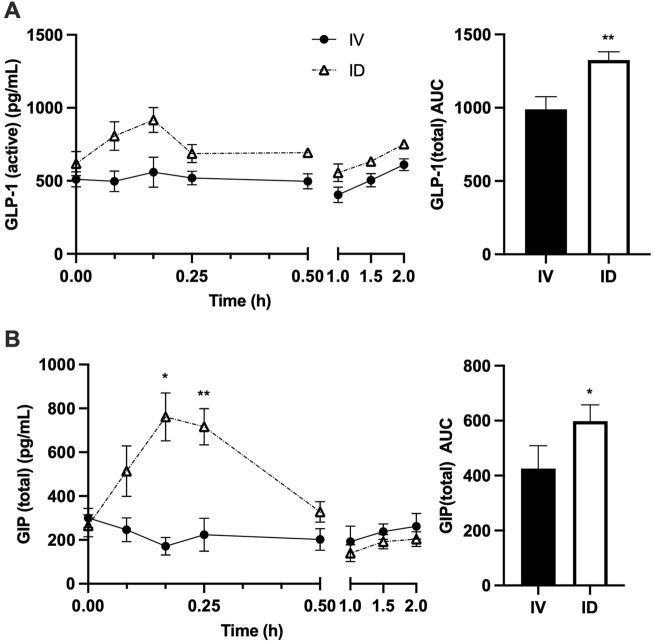
Intraduodenal (ID) and intravenous (IV) delivery of glucose lead to differences in circulating levels of incretin hormones. Serum concentrations (left) and AUC (right) of **(A)** GLP-1 and **(B)** GIP. Data are mean ± SEM. n = 7 each group. *p < 0.05, **p < 0.01 IV vs. ID.

As expected, serum glucose increased following both treatments; however, intravenous glucose infusion resulted in more rapid and dramatic increase in serum glucose, leading to significantly higher serum glucose at 5 min after treatments for IV vs. ID (*p* < 0.001) (Figure 3A). Serum insulin levels were not significantly different between treatments despite a trend to be higher in ID compared to IV after 30 min (Figure 3B), probably due to higher insulin secretion as reflected by higher C-peptide levels in ID vs. IV (*p* < 0.05 at both 30 and 90 min) (Figure 3C). Serum glucagon levels tended to be higher in ID compared with IV but did not reach statistical significance (Figure 3D). Serum GLP-1 levels tended to be higher following intraduodenal glucose as compared with intravenous glucose but did not reach statistical significance (Figure 4A). Since GLP-1 and GLP-2 are co-secreted in a 1:1 M ratio, GLP-2 (not measured) is expected to be affected similarly to GLP-1. Intraduodenal glucose rapidly increased serum GIP levels from baseline by more than two folds at 10 and 15 min before declining to baseline level thereafter (Figure 4B). In contrast, serum GIP levels remained stable following intravenous glucose. As a result, at 10 and 15 min after treatments, serum GIP levels were significantly higher in ID as compared with IV (*p* < 0.05 at 10 min, *p* < 0.001 at 15 min). A panel of other metabolic related hormones (secretin, PYY, PP, leptin, ghrelin and amylin) showed no or moderate differences between treatments (Supplemental Figure S1). These results indicate that the routes of glucose delivery were accompanied by differential changes in circulating glucose and several metabolic hormones. The areas under the curve (AUCs) for C-Peptide 2, glucagon, GLP-1 and GIP were all higher following intraduodenal vs. intravenous glucose. However, the most prominent difference was significantly higher circulating levels of GIP at timepoints corresponding to increased gut lipid output with intraduodenal glucose. It is well known that glucose in the GI track stimulates the secretion GIP, thus plasma GIP levels increase and correspond to plasm glucose levels during oral glucose tolerance test in mice (Pederson et al., 1998). Increases in GIP here also followed the rapid rise in glucose, supporting that intraduodenal glucose drives GIP as the mediator of increased lipid output.

### GIP mobilized lipid stores from the intestine

**FIGURE 5:**
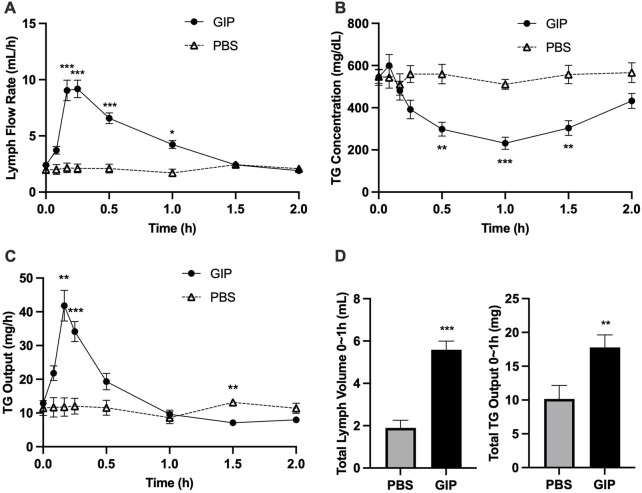
Intraperitoneal administration of GIP increases lymph fluid and TG output from the gut during post-absorptive state. **(A)** Lymph flow rate, **(B)** TG concentration and **(C)** TG output during the experiment, and **(D)** Total Lymph Volume and Total TG Output during the first hour. Data are mean ± SEM. n = 7 each group. *p < 0.05, **p < 0.01, ***p < 0.001 GIP vs. PBS.

**FIGURE 6:**
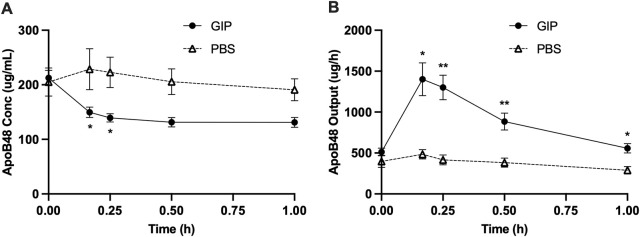
Intraperitoneal administration of GIP increases apolipoprotein B48 output from the gut during post-absorptive state. **(A)** Lymph ApoB48 concentration and **(B)** lymph apoB48 output during the experiment. Data are mean ± SEM. n = 7 each group. *p < 0.05, **p < 0.01 GIP vs. PBS.

In light of the dramatic increase in GIP around the timepoints of increased lipid output with intraduodenal glucose, we subsequently tested whether GIP *per se* stimulates intestinal lipid secretion. In this experiment, GIP was administered into the peritoneum 5 h following a lipid load, i.e., at exactly the same time when glucose was administered in the earlier study. Compared with control, GIP rapidly increased lymph flow rate by ∼3 folds at 10 and 15 min (*p* < 0.001 at 10, 15, 30 min, *p* < 0.05 at 60 min, GIP vs. PBS) (Figure 5A). GIP also caused significantly lower lymph TG concentrations compared with control (*p* < 0.01 at 30 and 90 min, *p* < 0.001 at 60 min, GIP vs. PBS) (Figure 5B). Despite more diluted lymph TG, TG output were significantly higher following GIP treatment compared with control (*p* < 0.01 at 10 min, *p* < 0.001 at 15 min) (Figure 5C). Total lymph fluid volume and total TG output were significantly higher within the first hour of GIP treatment compared with control (*p* < 0.001 and *p* < 0.01, respectively) (Figure 5D). Lymph ApoB48 concentrations were lower in GIP group than in PBS group, with values at 10 and 15 min reached statistical significance (*p* < 0.05 GIP vs. PBS at both timepoints) (Figure 6A). Lymph ApoB48 output increased from baseline following GIP but remained unchanged following PBS, resulting in ApoB48 output significantly higher in GIP than in PBS during 1 h post treatments (Figure 6B). These results clearly demonstrate robust effects of GIP in stimulating lipid secretion during the post-absorptive state.

## Discussions

The intestine not only digests and absorbs lipids during meal ingestion, but also retains part of dietary lipids into the post-absorptive state. The capacity for the intestine to store and later release lipids is linked to metabolic health, but the regulatory mechanisms remain poorly defined. Here we examined potential regulators of this process, taking advantage of a unique animal model, the mesenteric lymph duct cannulated rat. This model allows for direct collection of intestinal drainage to assess post-absorptive lipid release dynamics. We first reveal that intraduodenal delivery of glucose does not promote lipid secretion as does intravenous delivery of glucose. This difference was accompanied by differential changes in metabolic hormones, among which GIP exhibited the most striking difference between the two glucose delivery routes. We further establish that GIP *per se* promotes post-absorptive lipid release. These findings provide important insights into the regulation of lipid storage and release in the intestine.

Lipoprotein secretion in the intestine is known to respond to carbohydrates in intestinal lumen. In humans, concomitant infusion of glucose or fructose with lipids into the duodenum promoted lipoprotein biosynthesis (Stahel et al., 2019). In addition, oral glucose ingestion stimulated chylomicron secretion during the post-absorptive state (without luminal lipids) in humans (Xiao et al., 2019c) and rodents (Stahel et al., 2019). Release of intestinal lipid stores by oral glucose in humans involves mobilizing cytoplasmic lipid droplets towards chylomicron synthesis and secretion (Xiao et al., 2019c). While these studies support the role of luminal glucose as a trigger for the release of intestinally stored lipids, the exact mechanisms are not fully known. In rats, glucose uptake *via* glucose transporter and subsequent metabolism in enterocytes are required (Tian et al., 2022). Enterocytes are polarized cells and can also take up lipid and glucose substrates from the basolateral side. Intravenous infusion of glucose, which provides glucose at the basolateral side, stimulated chylomicron biosynthesis in humans (Xiao et al., 2016). One key question remains regarding how enterocytes are signaled to synthesize and secrete chylomicrons during the post-absorptive state; that is, do enterocytes discern apical vs. basolateral presence of glucose? Here we show that glucose provided *via* intravenous infusion does not stimulate chylomicron secretion during the post-absorptive state. This contrasts with post-prandial chylomicron biosynthesis and highlights different regulatory mechanisms between these two states. This finding supports a unique apical glucose sensing mechanism for the release of intestinal lipid stores. The underlying molecular mechanism is not clear but may be related to different glucose transporters on the apical and basolateral side of the enterocytes. Enterocytes express both glucose transporter 2 (GLUT2) and sodium-dependent glucose transporter 1 (SGLT1) at the apical membrane, but only GLUT2 at the basolateral membrane (Koepsell, 2020). To this end, the SGLT1 inhibitor phlorizin blocked luminal glucose mobilization of intestinal lipid stores (Tian et al., 2022). This points to a molecular link between SGLT1-mediated glucose uptake, intracellular glucose metabolism, and cytoplasmic lipid droplet mobilization towards chylomicrons synthesis. Such molecular mechanisms warrant further study.

By profiling key gluco-regulatory hormones in response to the two glucose delivery routes, we observed that several hormones were differentially affected. It is well known that glucose in the GI track stimulates the secretion of several gut hormones. For example, in mice, plasma GIP levels increase and correspond to plasm glucose levels during oral glucose tolerance test (Peterson 1998). In the current study, GIP levels were significantly higher in response to duodenal glucose. This occurred at timepoints corresponding to increases in glucose levels and gut lipid output, supporting that intraduodenal glucose drives GIP as the mediator of increased lipid output. Several other hormones were increased but to a lesser extent (e.g., secretin) or tended to increase but at later timepoints (e.g., leptin), suggesting that they are less likely to play significant roles in lipid mobilization. This prompted us to examine whether GIP is capable of releasing lipid stores in the intestine. Indeed, intraperitoneal GIP administration rapidly and robustly stimulated lipid output from the intestine, in a similar pattern to luminal glucose. To our knowledge, this is the first study to demonstrate a novel mechanism of regulation in the enterocytes where GIP modulates intestinal lipid handling in general and post-absorptive release of lipid stores from the intestine specifically. Since GIP administration decreased TG and apoB48 concentrations in lymph while intraduodenal glucose did not have such effects, GIP should not be interpreted as the sole mediator responsible of the effect of intraduodenal glucose. Future studies are required to tease out the role of GIP in the effects of intraduodenal glucose, such as with GIP receptor blockage.

How GIP exerts such effects is not known. One possibility is that GIP directly targets the enterocytes. GIP receptor mRNA is detected in rat duodenum and proximal small intestine epithelium (Usdin et al., 1993) and in mouse jejunum mucosa (Coon et al., 2013), although the exact cell types remain unclear (Hammoud and Drucker, 2023). In isolated mouse enterocytes and IEC-6 cells (a rat intestinal crypt-like epithelial cell line), GIP increases cAMP and stimulates glucose and peptide transport (Singh et al., 2008; Coon et al., 2015), indicating direct functional effects of GIP on enterocytes. A possibility cannot be ruled out that GIP indirectly affects chylomicron synthesis and secretion *via* unidentified intermediate cell type(s). Molecular mechanisms of action of GIP has been mostly studied in pancreatic beta cells (Muller et al., 2025). In pancreatic beta cells, glucose uptake into pancreatic beta cells is mediated by GLUT1 in humans and GLUT2 in rodents. GIP receptor activation in pancreatic beta cells increases cAMP levels, which in turn increases PKA/EPAC activity and Ca^2+^ influx to promote insulin granules exocytosis. Such molecular mechanisms have not been examined in intestinal cells. Examination of the expression of GIP receptor in specific intestinal cell types and molecular events downstream of its activation, such as roles of glucose transporters (e.g., GLUT2, SGLT1) and their involvement in lipid mobilization, would provide more mechanistic insights. If GIP mediated the effects of luminal glucose on intestinal lipid output, then a molecular mechanism can be envisioned to include glucose uptake *via* SGLT1, glucose metabolism, GIP secretion, cytoplasmic lipid droplet mobilization, and chylomicron synthesis and secretion. Although such a pathway and sequence of events cannot be established in this study, increased lipid output with exogenous GIP supports a link between GIP receptor activation and chylomicron secretion. Future research is needed to elucidate the molecular mechanisms of such gut-derived hormones in regulating lipid metabolism in enterocytes, through pharmacological inhibition or genetic ablation of GIP receptor. It is also worthwhile to investigate whether other hormones may have synergistic or antagonistic effects to GIP. Lastly, cautions should be exercised to extrapolate these findings to humans, considering species differences in GI physiology and gut hormone responses.

In summary, we demonstrate two important aspects of how lipids retained in the intestine is released during the post-absorptive state. Firstly, in contrast to glucose delivery into the intestinal lumen, glucose delivery to the blood circulation does not promote lipid output, pointing to a mechanism of glucose sensing at the lumen side. Secondly, GIP (an incretin hormone that is stimulated by luminal glucose for secretion) promotes lipid output, which for the first time demonstrates a role of GIP in lipid handling in the intestine. The latter point is with important clinical relevance, as the GIP/GLP-1 dual receptor agonist tirzepatide improves lipid profiles and cardiovascular risk biomarkers in patients with type 2 diabetes (Wilson et al., 2022; Pirro et al., 2022). Elucidating the mechanisms of intestinal lipid storage and release in metabolic diseases may help develop new strategies for the treatment and prevention of dyslipidemia and atherosclerotic cardiovascular diseases.

## Data Availability

The original contributions presented in the study are included in the article/Supplementary Material, further inquiries can be directed to the corresponding author.
