# Supplementary figures and images for: Glucose-dependent insulinotropic polypeptide stimulates post-absorptive lipid secretion in the intestine
Source: Front Physiol. 2025 Apr 4;16:1549392. doi: 10.3389/fphys.2025.1549392 (PMC12006050; doi:10.3389/fphys.2025.1549392)

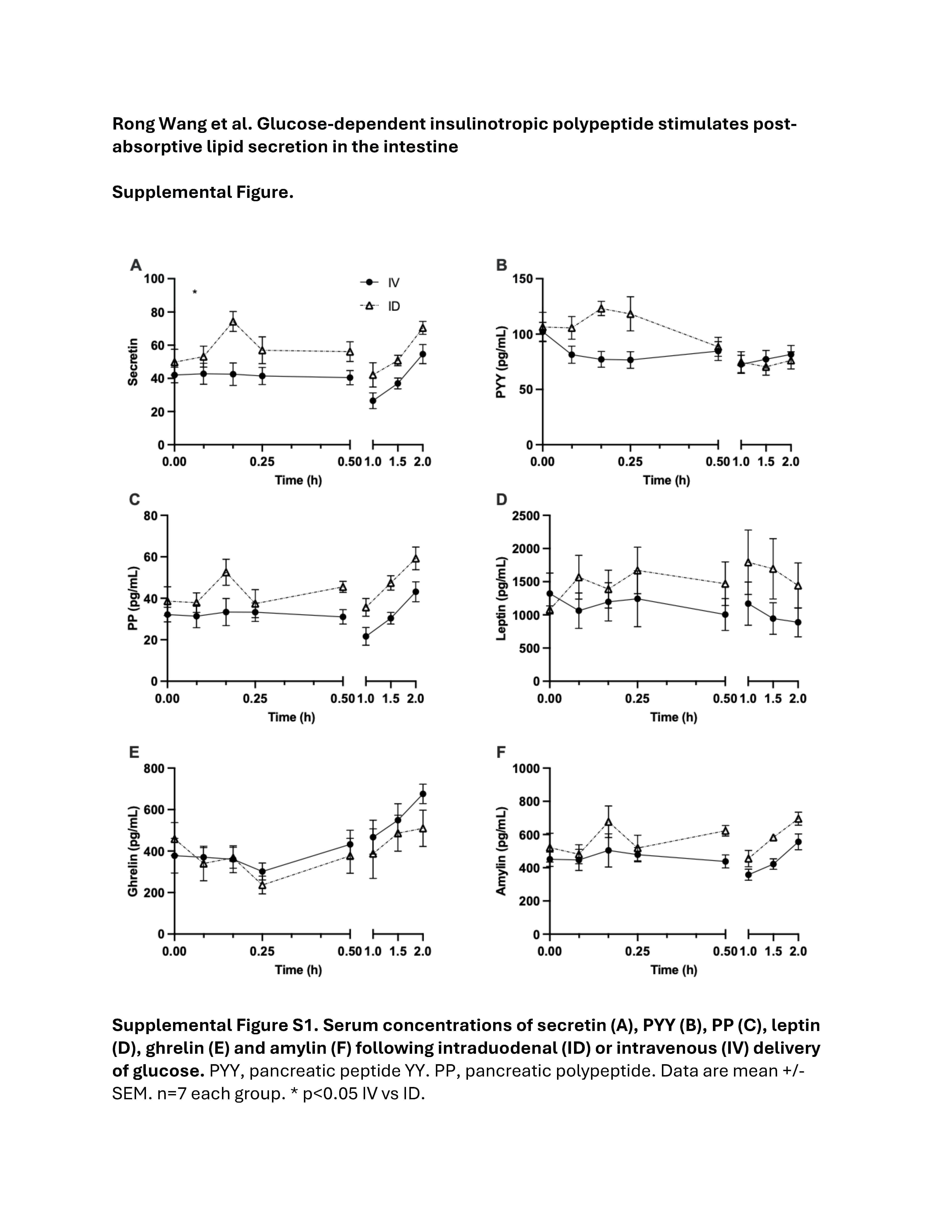

Supplement: Supplementary file 1 [file Image1.jpeg]
